# Seminar program for postgraduate specialty training in general practice: proposal for a 5-year thematic catalogue

**DOI:** 10.3205/zma001137

**Published:** 2017-11-15

**Authors:** Susanne Sommer, Erika Baum, Julia Magez, Jean-Francois Chenot, Gesine Weckmann, Jost Steinhäuser, Susanne Heim, Dagmar Schneider, Stephan Fuchs

**Affiliations:** 1 German College of General Practitioners and Family Physicians, Section of postgraduate training, Berlin, Germany; 2 Philipps-University Marburg, Department of General Practice, Preventative Medicine and Rehabilitation Medicine, Marburg, Germany; 3 Ruprecht-Karls-University Heidelberg, Department of General Practice and Department of General Practice and Health Services Research, Heidelberg, Germany; 4 University Medicine Greifswald, Institute for Community Medicine, Department of General Practice, Greifswald, Germany; 5 University Hospital Schleswig-Holstein, Campus Lübeck, Institute of Family Medicine, Lübeck, Germany; 6 University Medical Center of Göttingen, Department of General practice, Göttingen, Germany; 7 Coordination Center General practice, c/o Bavarian State Medical Association, München, Germany; 8 Martin-Luther-University Halle-Wittenberg, Department of General Practice and Family Medicine, Halle (Saale), Germany

**Keywords:** primary care, rotation network program, post graduate trainees, tutorial, learning, content

## Abstract

**Introduction**: In different German regions, seminar programs have been conducted for General practice residents. In each region, selection and teaching of learning content is conducted in a different manner. So far, no structured, standardized curriculum has been implemented nationwide. We have investigated, if the development of a common 5-year program of learning topics is conceivable between the different university departments of General practice in Germany.

**Method: **The seminar program working group of the DEGAM (German College of General Practitioners and Family Physicians) has conducted an online survey based on information gathered via preliminary telephone conference (n=7; physicians with postgraduate teaching experience) among all German university departments of General Practice and two non-university teaching institutions, identified via the internet. 884 topics were extracted from 14 Seminar programs. The topics were entered in a database, discussed and categorized: Practice management/practice work flow/standardized documentation forms/quality management (n=33 topics), common acute and chronic diseases, including disease management programs (n=29 topics), communication, neurological, psychological and psychiatric consultations (n=24 topics), common medical problems, including eye, ear, nose, throat, skin and pediatric problems (n=99 Topics) family physicians general approach, including epidemiology, shared decision making, test of time (n=42 Topics). These topics have been rated for priority and desirable number of teaching-units.

**Results:** A catalogue of 111 topics was designed, encompassing 160 teaching units. There is a suggestion of wide topics collections plus an add-on catalogue.

**Conclusion**: A proposal for a 5-year-thematic catalogue for postgraduate training of general practice residents in Germany has been developed. This newly developed curriculum has the potential to improve knowledge and skills that have not been covered during in-house and ambulatory general practice residencies.

## Zusammenfassung

**Einleitung:** An einigen Standorten in Deutschland werden Seminarprogramme für Ärzte in Weiterbildung zum Facharzt für Allgemeinmedizin (ÄiW) durchgeführt. Die Lerninhalte wurden bisher regional unterschiedlich ausgewählt. Ein deutschlandweit einheitlicher Themenkatalog fehlt. Ziel war es eine Themensammlung für ein 5-Jahre dauerndes Seminarprogramm zu entwickeln.

**Methode: **Die Arbeitsgruppe Seminarprogramme der DEGAM (Deutsche Gesellschaft für Allgemeinmedizin und Familienmedizin) hat auf Grundlage einer Telefonkonferenz (n=7 Ärzte mit Referentenexpertise) eine Online-Umfrage aller universitären allgemeinmedizinischen Abteilungen Deutschlands durchgeführt. Weitere zwei Anbieter wurden durch Internetrecherche detektiert. Aus 14 zurückgemeldeten Seminarprogrammen wurden 884 Themen zusammen getragen. Diese wurden in einer Datenbank eingepflegt, Doppelnennungen gestrichen und kategorisiert: Praxismanagement/Praxisablauf/Formularwesen/Qualitätsmanagement (n=33 Themen), häufige akute und chronische Erkrankung inclusive Disease Management Programme (n=29 Themen), Kommunikation, neurologische, psychische und psychiatrische Beratungsanlässe (n=24 Themen), häufige Beratungsanlässe/ inkl. aus „kleinen“ Fachgebieten: Auge/Haut/HNO/Pädiatrie (n=99 Themen), hausärztliche Arbeitsweisen (n=42 Themen). Anschließend wurden diese nach Wichtigkeit und Anzahl der Unterrichtseinheiten im Rahmen eines Expertenworkshops bewertet.

**Ergebnisse: **Es entstand ein Vorschlag für eine 160 Unterrichtsstunden (mit 111 Einzelthemen) umfassende Themensammlung mit einem Zusatzkatalog („Add-on“).

**Schlussfolgerung: **Es liegt für Deutschland eine vorläufige Version für einen bundesweit erarbeiteten Themenvorschlag für ein 5-Jahres-Seminarprogramm vor. Dadurch können die Weiterbildung begleitende Seminarprogramme standortübergreifend inhaltlich abgestimmt werden. Hierdurch ergibt sich potentiell die Möglichkeit Wissens- und Kompetenzlücken, die in der praktischen Tätigkeit der ambulanten und stationären Weiterbildungsabschnitte häufig verbleiben, zu reduzieren.

## Introduction

In Germany, postgraduate medical specialty training for the field of general practice and family medicine (GP) has a duration of 5 years, of which at least 18 months consist of an in-house residency in Internal Medicine and 24 months of ambulatory General Practice residency training. In addition, the GP trainees have to complete a 50 hours course of psychosomatic in primary care and 30 hours Balint-group attendance [http://www.bundesaerztekammer.de/fileadmin/user_upload/downloads/20130628-MWBO_V6.pdf]. Compared to other countries, there is significant need for improvement [1], [2]. Nationwide measures to enhance quality of postgraduate medical specialty training have so far proven to be insufficient [3]. Numerous structural recommendations have been proposed [4]. Accompanying seminar programs are only offered at a few locations. These were supposed to cover or enhance knowledge of topics that are underrepresented during postgraduate medical training. The German College of General Practitioners and Family Physicians (DEGAM) has proposed improvements for postgraduate medical specialty training. The DEGAM concept for improvement is called *DEGAM-Verbundweiterbildung^plus^* and envisions 32 annual teaching units by accompanying seminars [http://www.degam.de/weiterbildung.html]. The competence-based curriculum for general practice postgraduate medical training [https://www.weiterbildung-allgemeinmedizin.de/downloads/Curriculum_01-10-15.pdf] encompasses competencies that residents in postgraduate general practice training are expected to learn. In some German regions, seminar programs are offered by institutions like university departments, regional medical associations, coordination centers, postgraduate training networks. Several German states have already implemented structured, statewide seminar programs (Bavaria [http://www.kosta-bayern.de], Baden-Wuerttemberg [http://www.allgemeinmedizin-bw.de/startseite/], Hessen [http://www.allgemeinmedizin.uni-frankfurt.de/weiter/kolleg.html]). Less extensive postgraduate training courses and seminar programs for resident physicians are found in regions like Bremen, Göttingen, Greifswald, Halle, Schleswig-Holstein and Essen. In other regions of Germany, these programs are in the developmental phase.

Now, these seminars should be introduced nationwide. With the implementation of the Care Improvement Law (GKV-Versorgungsstärkungsgesetzes) [http://www.bmg.bund.de/themen/krankenversicherung/gkv-versorgungsstaerkungsgesetz.html], nationwide competence centers for postgraduate general practice training will be established that should be affiliated with university departments of General Practice [5]. The implementation of postgraduate training seminars will be mandatory (Annex IV of the framework agreement to Article 75a SBG V - GKV – Supply Enhancement Act [http://www.bmg.bund.de/themen/krankenversicherung/gkv-versorgungsstaerkungsgesetz.html]). This is another reason why the seminar programs of the five-year postgraduate general practice training will gain importance, which will serve to improve postgraduate training. A comprehensive, consented curriculum can provide important content-related impulses for new and existing seminar programs. These seminar programs can play an important role in the identity formation of future general practitioners [6] Specific knowledge, which is important for patient care in a general practice setting, will also be expanded [7]. The goal of the seminar programs is that the resident physician gains valuable knowledge and skills for working in general practice. In Germany, there is a tendency to compel general practitioners to license for elaborate supplementary qualifications after general practice training, for example in the area of geriatrics, pain therapy and palliative medicine [8]. These supplementary qualifications undermine the value of postgraduate training. It is necessary to develop a 5-year curriculum in order to prepare the resident physicians for essential consultation and treatment requirements of general practice and to enable them to reflect on their own actions. We aimed to create a consented collection of learning topics for these seminar programs, in cooperation with academic departments of GP, postgraduate teachers, coordination centers for postgraduate general practice training and resident physicians. We aimed to clarify if consensus could be reached on priorities and desirable number of teaching units of topics for seminar programs.

## Material / Methods

The list of topics was developed by mixed method design before it was reviewed and amended several times. The following methods were used: focus group discussions, online surveys about seminar programs, categorization of topics and World-Café [9].

### Telephone conference

Experienced organizers of seminar programs participated in a telephone conference. Seven physicians, most of whom also working as research associates at universities, took part. The participants also represented a large part of the author group of this article. In the course of their activities they regularly serve as postgraduate teachers, consultants or supervisory organizers in various general practice seminar programs.

Recruitment of seminar programs

We contacted all university institutions for general practice in Germany as well as the Bavarian Coordination Center for General Practice postgraduate training (KoStA) and forwarded a questionnaire to them about the content of their seminar programs. We received 12 responses from the general practice university departments [n=37] and from KoStA on behalf of the Bavarian universities. A second recruiting attempt was made via a pragmatic online research to identify non-university organized seminar programs.

#### E-mail Survey

In this survey, we requested a list of all topics previously covered in postgraduate training.

#### Data collection & categorization

All topics mentioned in the responses, were summarized in a database. The authors agreed to delete duplicate topics by consensus. Similar or content-related seminar topics were merged. The results were categorized into five groups. Nearly all topics were clearly categorized. The categories were predefined by consent in the initial telephone conference:

1. Practice management/practice work flow/standardized documentation forms/quality management (n=33)
2. Common acute and chronic diseases (n=29)
3. Communication, neurological, psychological and psychiatric consultations (n=24)
4. Common medical problems, including eye, ear, nose, throat, skin and pediatric problems (n=99)
5. General practitioner2.0/ GP’s general approach (n=42)

The differentiation between acute and chronic disease on the one hand and the reasons for consultation on the other was defined as follows: The first deals with the entire clinical picture. In the scope of reason for consultation, the patient presents a symptom which could be caused by various underlying diseases.

#### Assessing topics

During the telephone conference, it was also decided that the importance of implementing the individual seminar topics was to be rated on a scale of 0 to 3 points. A score of “0” equates to “not important” while a score of “3” indicates that the topic is “very important”. The number of teaching units was based on a Likert-scale rating from 0 to 5 (each teaching unit 45 minutes long). This rating was implemented in the course of a conference by the German College of General Practitioners and Family Physicians. That workshop was offered in the preliminary conference program and all conference attendees were able to register and attend the workshop. In the run-up to the conference, all departments for general practice in Germany were contacted and invited to the event. 25 subjects participated (physicians [n=21], including 3 resident physicians and non-medical research associates [n=4], there by representing a substantial part of the academic general practice in Germany. 

#### „World-Cafe“

A “World Café” was conducted with participants of the DEGAM workshop [9]. A table was prepared for each of the 5 topics (see above). Groups of 3-5 participants were seated at these tables and prompted to rate the various seminar topics with regard to their importance and the desired number of corresponding teaching units. The groups of participants moved from table to table every 20 minutes.

#### Data analysis

Afterwards, two transcribers transferred the paper-based results into digital forms and verified their consistency. For all topics, mean values with standard deviations were calculated for the characteristics “importance” and “number of teaching units”. Based on the mean values a ranking was created (importance: “very important” => “not important”; number of teaching units: 5 units => 0 units).

Topics, which were not rated (due to lack of time), were subsequently rated with “0 points” for importance and “0 number of teaching units” by the authors. This affected less than 25% of the assessments per item. The scope of the thematic catalogue encomprised 160 teaching units corresponding to 4 seminar days with 8 teaching units each, within 5 years of postgraduate GP training, which is consistent with the current DEGAM recommendations. Topics above the 160-teaching unit limit or new topics mentioned by the participants were compiled on the additional topic list “Add-on”.

## Results

Out of a total of 14 existing seminar programs, 884 topics were compiled. After the authors removed duplicates, 227 topics remained to be assessed at a later point. In case of duplications and very similar topics, academic GP experts most of them also engaged in postgraduate teaching, were consulted. Here from, a consented classification was performed. No discrepancies were identified.

In addition to the ratings according to “importance” and “number of teaching units”, workshop participants added personal, organizational, didactic notes to the worksheets. These comments will be evaluated and processed by a separate working group. Several participants suggested assigning the topics to a 2-year thematic catalogue and a 5-year thematic catalogue They reported that because of differences in funding of postgraduate general practice training between federal states, they would only be able to accommodate resident physicians in their seminar programs during ambulatory postgraduate training (approximately 2 years). We have so far not implemented this suggestion because the curriculum can be assembled in a modular way and can be differentiated by stakeholders on a regional level in consultation with the resident physicians (siehe attachment 1 ).

The newly created category “add-on” (see table 1 (Tab. 1)) describes previously unconsidered learning topics. These either emerged from suggestions by workshop participants or were not considered for the thematic catalogue because of their low rankings. Many of these additionally requested topics under “add-on” (for example, motivational discussion skills, traumatization, ECG assessment) are already covered by other entities mentioned above. On request of the participants, certain topics (for example, Balint group) in the category neurological and psychiatric consultation were not taken into account for the rankings and were instead added to the “add-on” category. These are topics covered in other compulsory postgraduate training curriculum (for example, psychosomatic primary care). The goal is to use existing resources effectively and to avoid duplication. Individual items were moved to another category after the World Café, because of content-related reasons.

## Discussion

A proposal for a 5-year thematic catalogue for postgraduate training of general practice residents has been presented. The content of existing seminar programs was summarized and newly weighted. Another innovative factor is the participation of general practice university institutions for the composition of the seminar programs. The German Medical Assembly had already linked the obligatory participation in structured courses of 240 hours with a 3-year mandatory postgraduate training for GPs. The individual regional medical boards adopted various postgraduate training programs in the years 1993 to 1998 [10]. These obligatory courses were discontinued when the postgraduate training duration was extended to 5 years. The position paper of the DEGAM in May 2011 demands that physicians who enter into general practice after previously training in another field, are required to attend a mandatory postgraduate training program of 120 hours in addition to specific work experience requirements. 60 hours should be done in face-to-face courses [11]. The idea of an extra-occupational seminar program is not new. However, this was the first time that all German university chairs and institutes of General Practice were invited to participate in the development of such a program. The main objective of this teaching content collection is to impart postgraduate GP residents the difference between a GPs work to the work of a specialist physician as well as to impart topics that are rarely addressed during postgraduate training.

The compiled list is highly relevant as well for safe guarding high quality primary care, as for teaching physicians in general practice postgraduate training, especially since many of the participants of the coordination process possess many years of experience in postgraduate teaching. For the long-term, the approach described here could represent a model for other postgraduate specialty training programs. 

We grouped the topics into five categories prioritizing them. This categorization may partly appear somewhat constructed and some were subsequently changed. We can certainly discuss about the classification and designation of the individual topics to the various groups but the stated topics and their weighting were paramount. The future organizers of seminar programs can thus individually plan specific topics in accordance with regional peculiarities. It became clear that certain aspects of the first category “practice management / practice work flow / standardized documentation forms / quality management” are of crucial importance for an establishment as a GP [12]. The category “family physicians general approach” includes seminar topics that convey the typical activities of a family physician. The teaching of these competencies naturally does not take place in the stationary postgraduate training sessions.

Because there is a close connection between formal postgraduate training curriculum and the right to bill for medical services, the political implication of a uniformly enforced curriculum is considerable. Seminar programs teach essential content of family medicine in a structured, understandable and potentially nationwide manner. In the process, they directly aim to improve the knowledge and skills of future general practitioners. For example, additional qualifications as geriatrics, pediatrics, prevention, pain therapy, palliative medicine and addiction medicine are integrated, for which specialist qualifications are increasingly being demanded [8], even though these are core competencies of a general practitioner. Thus, the previously described curriculum is also a valid argument against those who try to paint the requirement for such qualifications subsequent to the completed postgraduate training arguing that these are sub-specializations. We need an ever-present general practice “path” when selecting topics and lecturers. In this way, the seminar topics have to be conveyed to the resident physicians within a family doctor context. If trainers come from specialized fields, they must explicitly familiarize themselves with the topic-related general practice teaching material before starting. Ideally there should be a general practitioner at their side as a co-lecturer. 

The authors are aware that this is a matter of topic selection. We consider our compilation to be a recommendation. It is a supplement and a concretization to the competence-based curriculum [https://www.weiterbildung-allgemeinmedizin.de/downloads/Curriculum_01-10-15.pdf], [13] and the Vichtensteiner document (Competencies of family doctors in the general practice of the future) [14].

Didactic formats (seminar, workshop, examination techniques, group work, and presentation) will be coordinated with the content categories focusing always interactive teaching forms as well as practical exercises. In addition, active elements have to be incorporated to enable the resident physicians themselves to prepare and execute [7]. Depending to the local organizers, individual topics can be condensed into teaching blocks. This allows certain topics to be considered in a more complex and multi-field manner, f.i. issues that are related to symptoms of diseases can be easier combined. 

At the moment, seminar programs are a voluntary additional offer for resident physicians. Not all of them can be reached and take part. In longer-term, our goal is that all residents will undergo such a seminar program accompanying their postgraduate training. 

An evaluation of nationwide events in Germany is carried out every 3 years by DEGAM. The general practice departments are frequently the link between the university education and postgraduate training. They have a close contact to the future doctors during the studies. One of our next steps for our recommended “learning topics of general practice” will be to evaluate this content with regard to its feasibility. From an international viewpoint, regular extra-occupational seminars are standard [2]. Thus, the advantage for optimization is clear. The disadvantage results out of its resource-intense training measures. This must be periodically evaluated and adjusted. 

Considering the current situation, the new provision for continuing education for the specialist physician of general practice contains regularly scheduled seminars [http://www.bundesaerztekammer.de/fileadmin/user_upload/downloads/20130628-MWBO_V6.pdf]. Our thematic catalogue imps to do precisely that providing recommendations about the topics which should be implemented prioritizing them according to importance and time. The continuing education committee of GMA has drafted two important and forward-looking position documents [15] serving for the conceptual ongoing development of the postgraduate training in Germany. 

## Strengths and weaknesses

The professional orientation of general practices as well as the continuing education are actually discussed [16].One of the strengths of this work is the consensus reached in a large group of participants of medical experts. A strong focus on content is the result. According to regional characteristics, certain topics can be modified in their scope and detail. A possible distortion (most of the participants having postgraduate teaching experience as family doctors and are employees from university institutions, only a few physicians undergoing postgraduate training) may have occurred through the composition of the workshop participants. The learning topics will be made available to all providers of seminar programs after this study has been published. 

## Limitations

Unfortunately, not all participants were familiar with the method of the World Café. In particular during the first round, this resulted in not all topics being rated. Therefore, not all topics were considered in detail and included in the weighting. The calculated time of 20 minutes per category table was initially difficult. This will be amended for the next change. By staging a test run, participants could better be prepared for the World Café as a platform for discussion and decisions. This would reduce the number of “missings”. Unfortunately, only one third of the university departments in Germany participated in this project.

Seminar programs can also be beneficial for doctors in continuing education in other specialist fields; individual modules could be offered and used simultaneously. Our methodical approach in the preparation of the topic list for the seminar programs has its weaknesses. We are unable to currently resolve these. In some groups of the World Café, for example, not all topics could be evaluated for reasons of time, so that their weighting may have been inadvertently devalued. Three residents were directly involved in the rating process. As ongoing seminar programs are consistently evaluated by participant questionnaires, these results were also included in the assessment of the workshop participants. A consensus process should be implemented during the next revision process. The newly formed competence centers, and also their representatives of resident physicians should participate. The thematic catalogue presented here can thus serve as the basis for a seminar program for future specialist doctors of general practice in Germany.

## Competing interests

The authors declare that they have no competing interests. 

## Supplementary Material

Teaching topics for a 5-year accompanying postgraduate training seminar program, classified into predetermined categories: Assignment of the topics on the basis of their expected importance (from 0 = not important to 3 = very important) and the assigned teaching units (0 to 5 teaching units, each 45 minutes). MV = mean value, SD = standard deviation.

## Figures and Tables

**Table 1:**
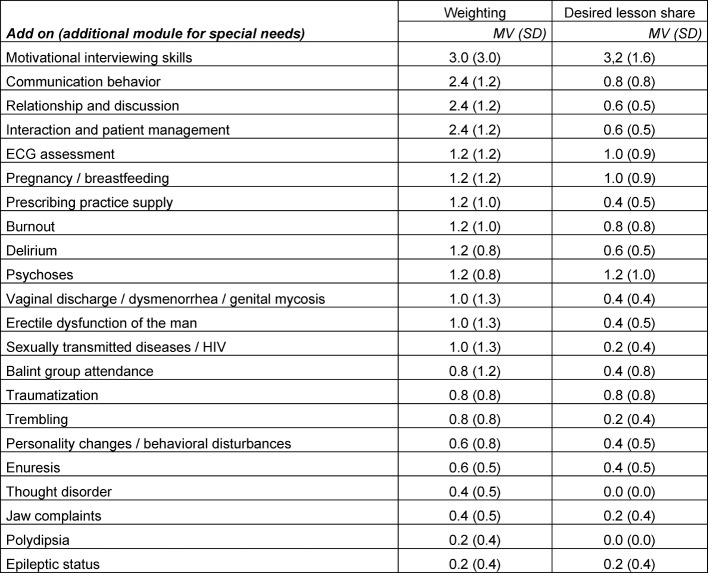
Additional teaching topics and subjects that can be optionally used / as well as topics that were not fully assessed
