# Supplementary material for: Seminar program for postgraduate specialty training in general practice: proposal for a 5-year thematic catalogue
Source: GMS J Med Educ. 2017 Nov 15;34(5):Doc60. doi: 10.3205/zma001137 (PMC5704617; doi:10.3205/zma001137)
Supplement: Teaching topics for a 5-year accompanying postgraduate training seminar program, classified into predetermined categories: Assignment of the topics on the basis of their expected importance (from 0 = not important to 3 = very important) and the assigned teaching units (0 to 5 teaching units, each 45 [file JME-34-60-s-001.pdf]

Attachment 1a: Topics: ***Practice management / practice work flow / standardized documentation forms/quality management***

Practice management/practice work flow/standardized documentation forms/quality management	Weighting	Desired lesson share
	MV (SD)	*MV (SD)*
Practice management	3.0 (0.0)	2.4 (1.5)
Technical examinations in general practice – what is necessary?	3.0 (0.0)	0.8 (0.8)
Founding a new practice	2.8 (0.4)	1.6 (1.0)
Team structure / daily life in the practice	2.8 (0.4)	1.4 (1.2)
Employment opportunities for general practitioners	2.6 (0.4)	1.0 (1.1)
Writing prescriptions, filling out sick leave forms	2.4 (1.2)	2.0 (1.7)
Scholarship and decision-making in patient care	2.4 (1.2)	1.4 (1.9)
Error management	2.4 (1.2)	1.0 (0.9)
Writing physiotherapy prescriptions	2.4 (1.2)	0.2 (0.4)
Prescribing assistive tools and devices	2.2 (1.2)	1.0 (1.3)
Personal time management / work-life balance / compatibility of family and career	2.2 (1.2)	1.0 (1.3)
Submitting rehabilitation requests	2.0 (1.1)	0.6 (0.8)
Responsibilities of the Association of Statutory Health Insurance Physicians/Medical Association	1.8 (1.2)	0.8 (0.8)
Crisis management	1.8 (1.2)	0.6 (0.8)
Ambulatory cooperation opportunities / practice models	1.8 (1.2)	0.6 (0.5)
Legal questions / legal aspects / practice management	1.6 (1.4)	0.8 (0.8)
Pharmaceutical guidelines	1.6 (1.2)	0.6 (0.8)
Request short-term care	1.4 (1.4)	0.6 (0.8)
Legal guidelines of a patient's advance and care directive	1.4 (1.2)	0.4 (0.5)
Avoid billing problems	1.4 (1.0)	0.2 (0.4)

Attachment 1b: Topics: Common acute and chronic diseases

Common acute and chronic diseases	Weighting	Desired lesson share
	MV (SD)	*MV (SD)*
Rheumatological diseases	3.0 (0.0)	2.0 (1.5)
Diabetes mellitus (hypo- / hyperglycemia)	2.8 (0.4)	2.2 (1.6)
Apoplexy/ Cerebrovascular disease	2.8 (0.4)	2.2 (1.0)
Gastritis / ulcer / reflux / esophagitis	2.8 (0.4)	1.8 (1.2)
Bronchial asthma	2.8 (0.4)	1.8 (0.8)
COPD	2.8 (0.4)	1.4 (0.8)
Hypertension	2.6 (0.8)	2.2 (1.0)
Disease of the kidney	2.6 (0.8)	1.6 (0.4)
Cardiac insufficiency	2.6 (0.8)	1.4 (0.8)
Coronary heart disease	2.6 (0.8)	1.4 (0.8)
Varicose veins / ulcus cruris venosum / thrombosis	2.6 (0.5)	2.2 (1.2)
Diabetic foot syndrome	2.6 (0.5)	1.8 (1.2)
Further rheumatic diseases	2.4 (1.2)	1.6 (1.7)
Thyroid diseases	2.4 (0.8)	1.8 (0.4)

Osteoporosis	2.2 (0.8)	1.2 (0.4)
Sleep apnea syndrome	2.2 (0.8)	1.0 (0.6)
Peripheral arterial occlusive disease	2.2 (0.8)	1.0 (0.6)
Heart rhythm disturbances	1.8 (1.2)	1.0 (0.9)
Prostate hyperplasia	1.8 (0.8)	1.0 (0.6)

Attachment 1c: Topics: *Communication, neurological, psychological and psychiatric consultations*

	Weighting	Desired lesson share
Neurological and psychiatric consultations	*MV (SD)*	*MV (SD)*
The difficult patient	2.8 (0.4)	1.0 (0.6)
Delivering bad news	2.6 (0.8)	1.0 (0.6)
Addiction in general practice	2.4 (1.2)	2.2 (1.6)
Depression	2.4 (1.2)	1.8 (1.3)
Dementia	2.4 (1.2)	1.8 (1.3)
Sleep disturbance	2.4 (1.2)	1.0 (0.6)
Anxiety and panic disorder	2.2 (1.2)	1.0 (0.9)
Paralysis and sensory disorder	2.2 (1.0)	1.4 (0.5)
Somatization disorder	1.8 (1.5)	2.4 (1.9)
Convulsion / cramps	1.6 (1.2)	0.4 (0.5)
Culturally induced social problems	1.8 (1.2)	1.6 (1.4)
Memory disorders/ concentration disturbance	1.4 (1.4)	0.2 (0.4)

Attachment 1d: Topics: *Common medical problems including minor problems*

	Weighting	Desired lesson share
Common medical problems including minor problems	*MV (SD)*	*MV (SD)*
Stomach ache / nausea	3.0 (0.0)	2.8 (1.2)
Back pain	3.0 (0.0)	2.6 (1.5)
Chronic pain	3.0 (0.0)	2.4 (1.3)
Chest pain	3.0 (0.0)	2.0 (1.1)
Cough	3.0 (0.0)	1.6 (1.2)
Shortness of breath	3.0 (0.0)	1.2 (0.4)
Dizziness	2.8 (0.4)	2.2 (1.2)
Chronic wounds	2.8 (0.4)	2.2 (0.8)
Incontinence / chronic bladder weakness	2.6 (0.8)	1.4 (0.5)
Tiredness	2.6 (0.5)	1.2 (0.5)
Acute pain	2.4 (1.2)	0.4 (0.5)
Threat to a child's welfare	2.2 (1.2)	1.8 (2.0)
Earache / facial pain	2.4 (0.8)	2.2 (1.0)
Acute infection / fever	2.4 (0.8)	1.8 (0.8)
Anal complaints	2.4 (0.8)	1.2 (0.4)
Syncope / loss of consciousness	2.2 (1.0)	1.2 (0.4)
Acute injuries	2.2 (0.8)	1.6 (0.5)
Skin diseases: itchiness / unclear skin changes	2.0 (1.3)	2.3 (1.3)

Developmental disorder in childhood	2.0 (1.3)	1.6 (1.9)
Burning when urinating	2.0 (1.0)	1.0 (1.0)
Diarrhea	2.0 (0.9)	1.0 (0.6)
The red eye / impaired vision	1.8 (1.5)	1.6 (1.0)
Bloody secretion / hemoptysis	1.8 (1.5)	0.4 (0.5)
Peculiar hemogram / increased liver values	1.8 (1.2)	1.2 (0.8)
Loss of voice / hoarseness	1.8 (1.2)	0.8 (0.8)
Joint pain / joint swelling	1.8 (1.2)	0.4 (0.5)
Uncertain lymph node swelling	1.8 (1.0)	1.4 (0.5)
Swollen leg / ankle edemas	1.8 (0.8)	1.2 (0.4)
Nosebleed	1.6 (1.5)	0.2 (0.4)
The sick child	1.6 (1.4)	0.8 (1.0)
Restless legs	1.6 (1.0)	0.8 (0.8)
Complaints of the mouth, tongue and lips	1.4 (1.0)	1.0 (0.6)
Heart: palpitations	1.4 (1.0)	0.6 (0.5)
Swallowing disorders	1.4 (0.8)	1.0 (0.6)
Skin Ulcers	1.2 (1.5)	0.4 (0.5)

Attachment 1e*: General Practitioner 2.0/ General practitioner work flow*

Topics: General Practitioner 2.0/ General practitioner work flow	Weighting	Desired lesson share
	MV (SD)	*MV (SD)*
Pharmacotherapy for the elderly	3.0 (0.0)	3.0 (1.3)
Polypharmacy/ medication management	3.0 (0.0)	2.0 (1.4)
Geriatric assessment / falls	3.0 (0.0)	1.6 (0.8)
Examination shoulder joint	2.6 (0.8)	1.6 (1.4)
Home visits	2.4 (1.2)	0.8 (1.0)
Guidelines	2.4 (1.2)	0.6 (0.8)
Preventive services for children and adolescents	2.2 (2.2)	1.2 (0.8)
Cancer diseases in General Practice	2.2 (0.8)	2.0 (1.7)
Examination hip, knee	2.0 (1.6)	1.8 (1.7)
Patient in a nursing home	2.0 (1.3)	1.6 (1.3)
Sexuality and family planning	2.0 (1.1)	0.6 (0.8)
Death certificate	1.8 (1.5)	1.2 (1.5)
Vaccination	1.8 (1.5)	1.2 (1.2)
Individual health services	1.8 (1.5)	0.8 (0.8)
Palliative medicine / terminal care / grief work	1.8 (1.2)	1.6 (1.0)
Prevention	1.6 (1.4)	2.2 (2.3)
Documentation in General Practitioner’s office	1.6 (1.4)	1.0 (0.9)
Reanimation in General Practitioner’s office	1.6 (1.4)	1.0 (0.9)
Diet, overweight / eating disorders / food intolerance	1.6 (1.2)	1.2 (0.8)
Domestic violence	1.4 (1.2)	1.2 (1.2)
Patient oriented evidence-based research	1.4 (1.2)	1.0 (0.9)
Legal representative for health care	1.4 (1.2)	0.8 (0.8)
Menopausal complaints of the woman	1.4 (1.2)	0.8 (0.8)

Specialist physician examination / prerequisite / application / process	1.2 (1.5)	0.6 (0.8)
Labor law questions from doctors in training	1.2 (1.5)	0.4 (0.5)

Attachment 1f: Additional teaching topics and subjects that can be optionally used / as well as topics that were not fully assessed

Add on (additional module for special needs)	Weighting	Desired lesson share
	MV (SD)	*MV (SD)*
Motivational interviewing skills	3.0 (3.0)	3,2 (1.6)
Communication behavior	2.4 (1.2)	0.8 (0.8)
Relationship and discussion	2.4 (1.2)	0.6 (0.5)
Interaction and patient management	2.4 (1.2)	0.6 (0.5)
ECG assessment	1.2 (1.2)	1.0 (0.9)
Pregnancy / breastfeeding	1.2 (1.2)	1.0 (0.9)
Prescribing practice supply	1.2 (1.0)	0.4 (0.5)
Burnout	1.2 (1.0)	0.8 (0.8)
Delirium	1.2 (0.8)	0.6 (0.5)
Psychoses	1.2 (0.8)	1.2 (1.0)
Vaginal discharge / dysmenorrhea / genital mycosis	1.0 (1.3)	0.4 (0.4)
Erectile dysfunction of the man	1.0 (1.3)	0.4 (0.5)
Sexually transmitted diseases / HIV	1.0 (1.3)	0.2 (0.4)
Balint group attendance	0.8 (1.2)	0.4 (0.8)
Traumatization	0.8 (0.8)	0.8 (0.8)
Trembling	0.8 (0.8)	0.2 (0.4)
Personality changes / behavioral disturbances	0.6 (0.8)	0.4 (0.5)
Enuresis	0.6 (0.5)	0.4 (0.5)
Thought disorder	0.4 (0.5)	0.0 (0.0)
Jaw complaints	0.4 (0.5)	0.2 (0.4)
Polydipsia	0.2 (0.4)	0.0 (0.0)
Epileptic status	0.2 (0.4)	0.2 (0.4)
